# Dosimetric impact of the respiratory motion of the liver dome in stereotactic body radiotherapy for spine metastasis: A planning study

**DOI:** 10.1002/acm2.14403

**Published:** 2024-07-01

**Authors:** Hiroyuki Okamoto, Midori Nonaka, Takahito Chiba, Tomoya Kaneda, Yuta Kobayashi, Satoshi Nakamura, Hiroki Nakayama, Kotaro Iijima, Yasunori Shuto, Miki Yonemura, Riki Oshika, Hironori Kishida, Yuka Urago, Masato Nishitani, Shuka Nishina, Takumi Sakamoto, Yoshihiro Shibata, Tomonori Goka, Hiroshi Igaki

**Affiliations:** ^1^ Radiation Safety and Quality Assurance Division National Cancer Center Hospital Tokyo Japan; ^2^ Department of Radiological Technology National Cancer Center Hospital Tokyo Japan; ^3^ Department of Radiation Oncology National Cancer Center Hospital Tokyo Japan; ^4^ Department of Radiation Oncology Juntendo University Graduate School of Medicine Tokyo Japan; ^5^ Department of Radiological Sciences Graduate School of Human Health Sciences Tokyo Metropolitan University Tokyo Japan; ^6^ Department of Radiological Sciences Komazawa University Graduate School Tokyo Japan

**Keywords:** dosimetric impact, SBRT, spine metastasis

## Abstract

**Purpose:**

This study aimed to clarify the dosimetric impact of the respiratory motion of the liver on stereotactic body radiation therapy (SBRT) for spine metastasis and examine the utility of introducing beam avoidance (beam‐off at specific gantry angles).

**Methods:**

A total of 112 consecutive patients who underwent SBRT for spine metastasis between 2018 and 2024 were examined. Overall, 15 patients who had lesions near the liver dome were included in this study. Retrospective treatment plans were generated using computed tomography (CT) images acquired during inhalation and exhalation to evaluate the dosimetric impact of respiratory motion of the liver. The dose difference (*DD)* and relative value (*DD*%) were evaluated using the dose‐volume histogram (DVH) metrics, planning target volume *D*_max_, *D*_95%_, spinal cord *D*_0.035 cc_, and esophagus *D*_2.5 cc_. The magnitude of the liver movements was evaluated based on differences of liver size *L*_ave_ at the isocentric axial plane between the inspiratory and expiratory CT images.

**Results:**

The *DD* in almost all DVH metrics tended to increase when the liver moved away from the target during inhalation: For example, Mean ± a standard deviation (SD) *DD* in PTV *D*_95%_ for the treatment plan incorporating beam avoidance and those without beam avoidance was 0.5 ± 0.3 and 0.9 ± 0.6 Gy, respectively. The spinal cord *D*_0.035 cc_ for those shows 0.4 ± 0.2 and 0.7 ± 0.7 Gy, respectively. The treatment plans without beam avoidance also showed moderate or strong correlations between *L*_ave_ and *DD* for almost all DVH metrics. No correlation was seen in the beam avoidance plan. The spinal cord *D*_0.035 cc_ revealed approximately 1 Gy or +4% in *DD* when *L*_ave_ was < −4 cm.

**Conclusions:**

Respiratory motion of the liver dome can cause substantial dosimetric discrepancies in the dose delivered to the spinal cord, although the extent depends on patient variables. Dose assessment should be performed for determining the appropriate means of respiratory management, such as breath‐hold. Alternatively, beam avoidance effectively mitigates the impact.

## 1 INTRODUCTION

Stereotactic body radiation therapy (SBRT) is an advanced^1^, ^2^ and improved radiotherapeutic approach to conform dose distribution to the limited target volume. Irradiation techniques such as intensity‐modulated radiotherapy (IMRT) allow the delivery of higher fraction doses with small treatment fractions. Additionally, image guidance before daily treatments can precisely localize the target. Higher biologically effective doses can be delivered compared with conventional radiotherapy, such as three‐dimensional radiotherapy (3DCRT), leading to improved clinical outcomes. The application of SBRT to various treatment sites, including the lung, liver, and prostate, has demonstrated clinical advantages.^3^, ^4^, ^5^ Recently, improved overall survival rates have been demonstrated in the treatment of oligometastasis including spine using SBRT.^6^

SBRT is commonly performed for lung and liver cancer with appropriate respiratory management.^7^, ^8^ The determination of the type of respiratory management including the breath‐hold technique or gated radiotherapy, depends on patient‐specific variables (such as capability, respiratory stability, and the magnitude of tumor movement), clinically requirement, and treatment efficiency. Unless there is a specific reason, respiratory management is not performed with SBRT for spine metastasis to the thoracic vertebrae. If a treated target is located near the liver dome, the respiratory motion of the liver may affect the dose distribution by changing the beam path of the liver during irradiation. In such cases, respiratory management should be considered for improving treatment accuracy. However, the dosimetric impact of respiratory motion of the liver remains unclear, and institutional policies vary. To date, little investigation has been done for its impact. Although respiratory management is important, it can be a burden on the patient and prolong treatment duration.

This study aimed to clarify the dosimetric impact of respiratory liver movement during SBRT for spine metastasis. We also examined the effectiveness of avoidance angles without beam‐on in volumetric modulated arc therapy (VMAT) for minimizing such impact.

## 2 METHODS

### 2.1 Patient characteristics and radiotherapy

**FIGURE 1:**
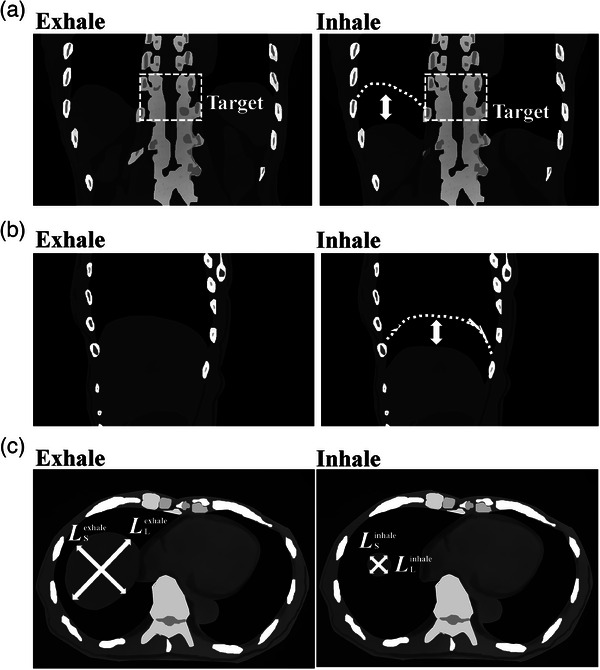
Illustration of respiration‐induced liver movement on (a) the coronal, (b) the sagittal, and (c) the axial plane.

Overall, 112 consecutive patients who underwent SBRT for bone metastasis with a fractional dose ≥6 Gy between 2018 and 2024 and had 208 treated lesions between them were retrospectively examined. Of these, 29 spine metastases were located near the liver dome. However, eight cases were excluded because they had only expiratory CT images and therefore could not be evaluated. In five cases, the liver dome did not move up and down in the isocentric axial plane. This meant that the effect of changing attenuation (lung and liver) on the beam path was negligible; therefore, these five cases were also excluded. Finally, 16 cases (15 patients, 1 patient had 2 lesions) were included in the study. Table 1 summarizes the patient characteristics and treatment information. The Varian TrueBeam radiation therapy system (Varian Medical Systems, Palo Alto, CA, USA) and the Varian Eclipse v. 16.1 treatment planning system (TPS) were used in this study. If the treatment site was at or near the level of the liver dome (as illustrated in Figure 1), two CT scans, one inspiratory and one expiratory, taken during inhaled and exhaled breath‐holds were acquired. The scans were transferred to the Varian Eclipse for treatment planning. Our institution has used the expiratory CT images as the primary CT images in the treatment planning for a long time, which is used for delineation and dose calculation. An isotropic geometrical margin of 2−3 mm to planning target volume (PTV) was applied. A medical physicist responsible for the patient's treatment planning was the planner in each case. Beam avoidance was implemented by the planner when it was deemed possible to effectively reduce the lung or liver dose or, occasionally, to keep the beam path away from the arm or liver. Lack of existing explicit criteria for such planning motivated us for this study. However, we adhered to the dose–volume histogram (DVH) criteria established in previous literature,^9^, ^10^, ^11^, ^12^, ^13^ as presented in Table 2.

**TABLE 1:** Patient characteristics and treatment information.

Case #	Prescribed dose	Site	Breathing during treatment	Beam avoidance for a clinically accepted treatment plan	Gantry angle range	Avoidance angle
1	35 Gy/5 Fr	Th10	Free	Off	Arc1: CW: 181−179° Arc2: CCW: 179−181°	Arc1: 265−345° Arc2: 345−265°
2	30 Gy/5 Fr	Th7	Free	On*^1^	Arc1: CW: 181−0° Arc2: CW: 0−179° Arc3: CCW: 179−181 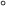	Arc1: 265−300° Arc2: 60−100° Arc3: 110−60°, 320−265°
3	35 Gy/5 Fr	Th11	Free	On*^2^	Arc1: CW: 181−179° Arc2: CCW: 179−181°	NA
4	35 Gy/5 Fr	Th9	Exhalation	On*^1^	Arc1: CW: 181−179° Arc2: CCW: 179−181°	Arc1: 260−305°, 70−120° Arc2: 120−70°, 305−260°
5	30 Gy/5 Fr	Th10	Free	On*^1^	Arc1: CW: 181−179° Arc2: CCW: 179−181°	Arc1: 260−290°, 80−120° Arc2: 120−80°, 290−260°
6	30 Gy/5 Fr	Th12	Free	On*^1^	Arc1: CW: 181−179° Arc2: CCW: 179−181°	Arc1: 260−290° Arc2: 290−260°
7	35 Gy/5 Fr	Th9	Free	On*^1^	Arc1: CW: 181−179° Arc2: CCW: 179−181°	Arc1: 230−320°, 60−95° Arc2: 95−60°, 300−265°
8	35 Gy/5 Fr	Th10, Th11	Exhalation	Off	Arc1: CW: 181−179° Arc2: CCW: 179−181°	Arc1: 260−325° Arc2: 325−260°
9	35 Gy/5 Fr	Th12	Free	Off	Arc1: CW: 181−179° Arc2: CCW: 179−181°	Arc1: 270−340° Arc2: 340−270°
10	35 Gy/5 Fr	Th10	Free	On*^1^	Arc1: CW: 181−179° Arc2: CCW: 179−181° Arc3: CW: 181−179°	Arc1: 270−90° Arc2: 90−270° Arc3: 270−90°
11	35 Gy/5 Fr	Th8	Free	Off	Arc1: CW: 181−179° Arc2: CCW: 179−181°	Arc1: 265−330° Arc2: 330−265°
12	35 Gy/5 Fr	Th7, Th8, Th9	Free	On*^1^	Arc1: CW: 181−179° Arc2: CCW: 179−181°	Arc1: 260−20° Arc2: 20−260°
13	40 Gy/5 Fr	Th9	Free	On*^1^	Arc1: CW: 181−179° Arc2: CCW: 179−181°	Arc1: 240−290° Arc2: 290−240°
14	35 Gy/5 Fr	Th12	Exhalation	Off	Arc1: CW: 181−179° Arc2: CCW: 179−181°	Arc1: 250−330° Arc2: 330−250°
15	35 Gy/5 Fr	Th10	Exhalation	On*^1^	Arc1: CW: 181−179° Arc2: CCW: 179−181°	Arc1: 250−285°, 70−105° Arc2: 105−70°, 285−250°
16	35 Gy/5 Fr	Th11	Exhalation	On*^1^	Arc1: CW: 181−179°	Arc1: 270−340°

*Note*: There were 15 patients but 16 cases as 1 patient had 2 lesions (cases 5 and 6).

Abbreviations: CCW, counterclockwise; CW, clockwise; Fr, fractions; Gy, Gray; Off, beam avoidance off; On^*1^, gantry‐based beam avoidance on; On^*2^, structure‐based beam avoidance on; Th, thoracic vertebrae.

**TABLE 2:** DVH criteria for radiation therapy planning.

Organ	Criterion	Goal
PTV*	*D*_95%_	–
Spinalcord_02 (PRV 2 mm)	*D*_0.035cc_	≤28 Gy
Esophagus	*D*_2.5cc_	≤27.5 Gy
Stomach	*D*_max_	≤32 Gy
Bowel	*D*_max_	≤29 Gy
Liver	*D*_mean_	–
	*D*_700cc_	≤21 Gy

Abbreviations: *D*, dose; DVH, dose‐volume histogram; PRV, planning at risk volume; PTV, planning target volume; PTV*, a reduction in the PTV margin allowed to meet the criteria, especially for the spinal cord and esophagus; spinalcord_02 (PRV 2 mm), spinal cord with a PRV margin of 2 mm.

### 2.2 Retrospective planning study

**FIGURE 2:**
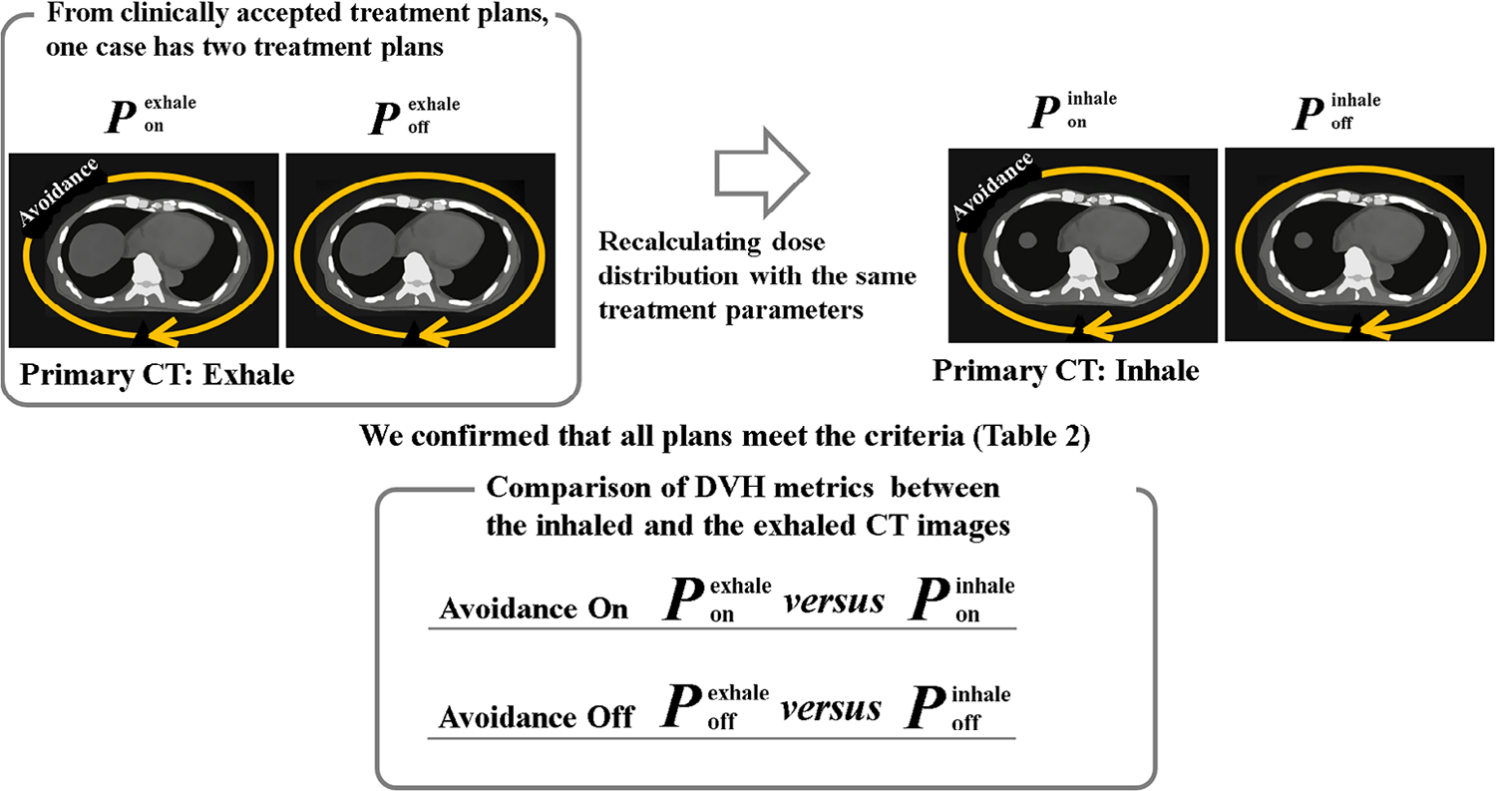
The design of a retrospective planning study for evaluation of the dosimetric impact of respiration‐induced liver movement.

**FIGURE 3:**
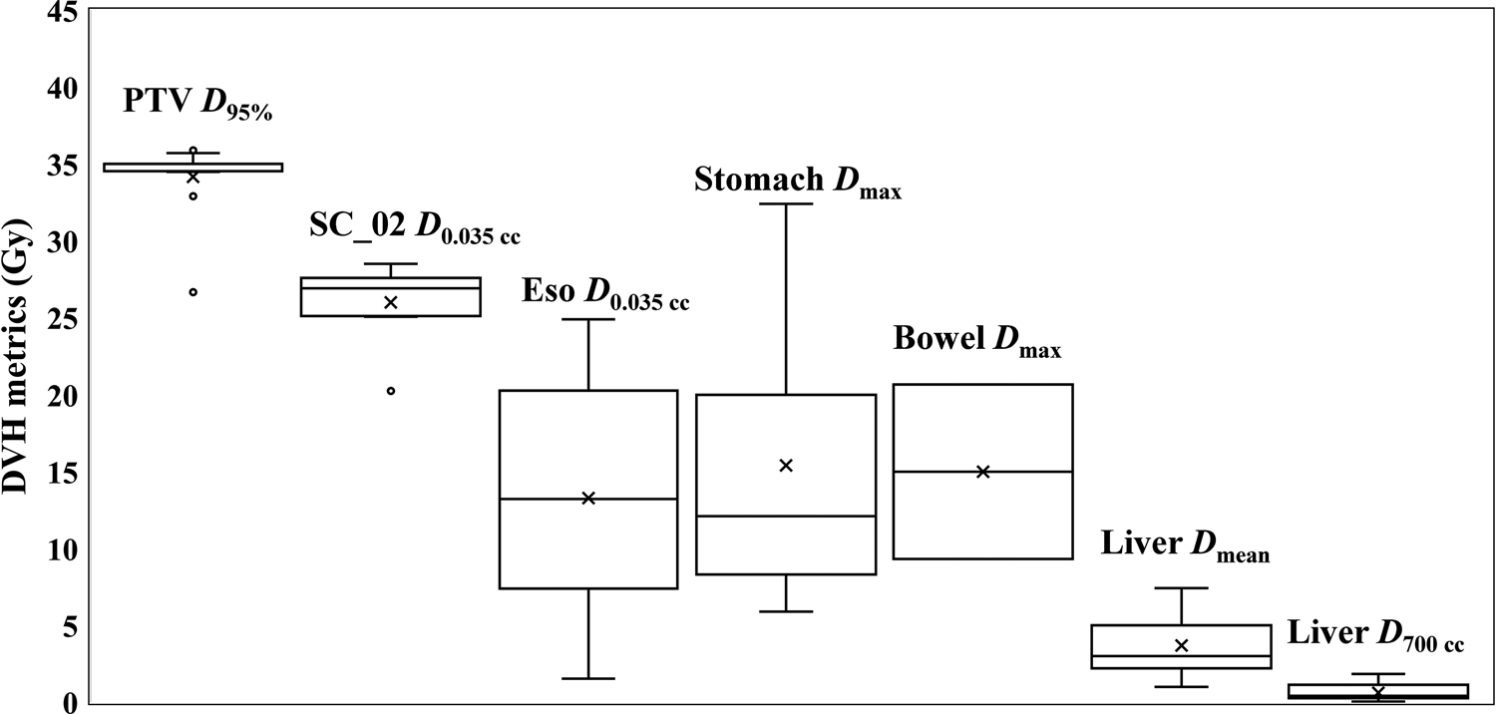
DVH metrics for the clinically accepted SBRT treatment plans used in this study. DVH, dose–volume histogram; Eso, esophagus; PTV, planning target volume; SBRT, stereotactic body radiation therapy; SC_02, spinal cord with a 2 mm geometrical margin. The *D*_95_ for patients 2, 5, 6, and 13 were excluded because the prescribed dose was not 35 Gy/5 Fr.

**FIGURE 4:**
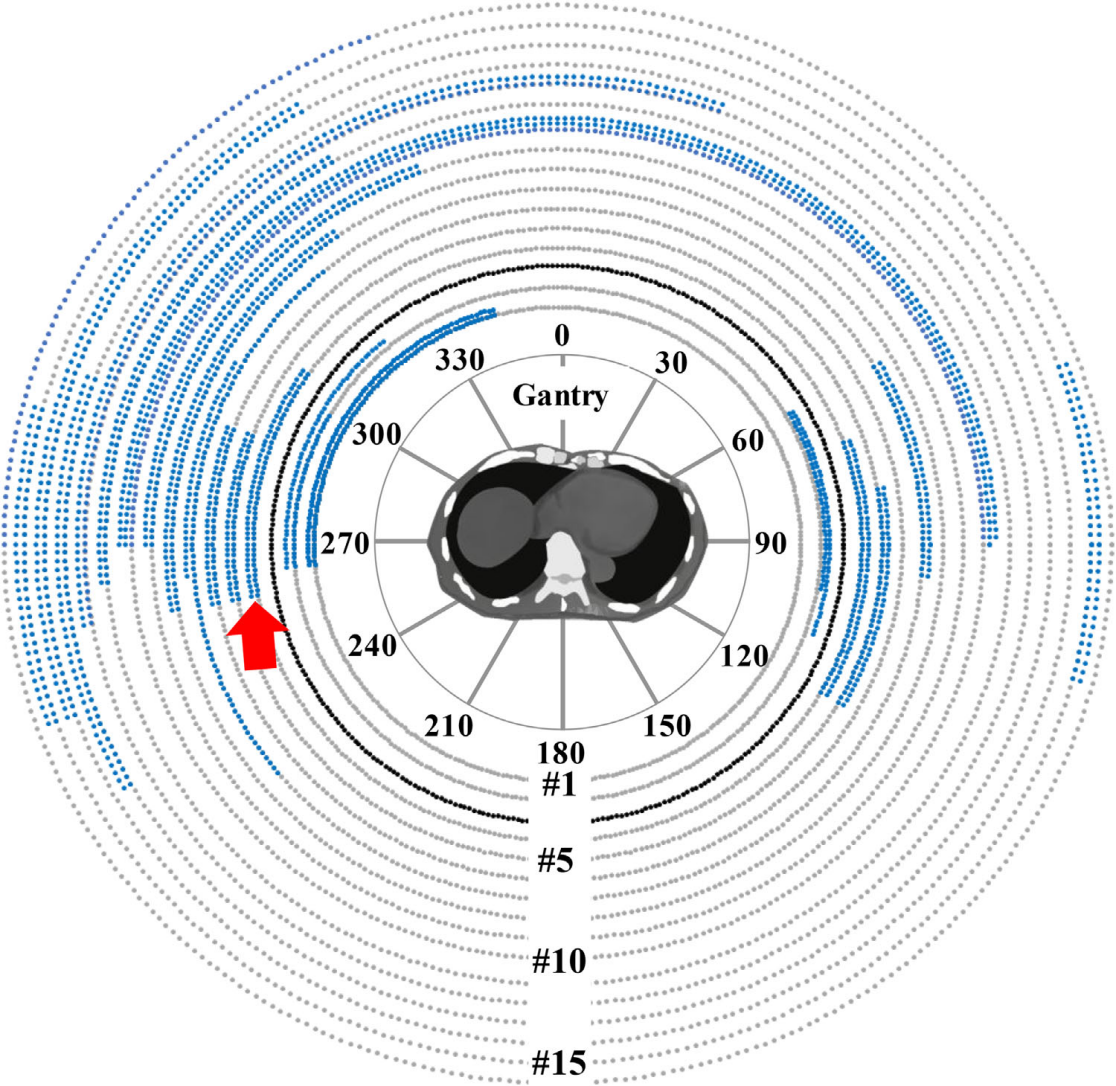
Illustration of the gantry angles determined for beam avoidance in all $P_{\mathrm{on}}^{\mathrm{exhale}}$ SBRT treatment plans. The structure‐based beam avoidance was used for the patient #3 (black line). SBRT, stereotactic body radiation therapy.

Design of this planning study is shown in Figure 2. We retrospectively examined 16 clinically accepted treatment plans. Figure 3 shows the DVH metrics for these treatment plans. These plans included the two plans with and without beam avoidance (BA), which is named $P_{\mathrm{on}}^{\mathrm{exhale}}$ and $P_{\mathrm{off}}^{\mathrm{exhale}}$, respectively. Varian Eclipse provides BA optimization functions that utilize either specific gantry angles or structure‐based constraints. Thus, in the presence of irradiation, the BA is based on a specific range of gantry angles or on the beam passing to specific organs. BA is often used in clinical practice to reduce the dose to an organ at risk (OAR), such as the lens, lung, or kidney. As shown in Table 1, 5 of the clinical treatment plans did not use BA, 10 used gantry‐based BA, and 1 used structure‐based BA. To retrospectively create $P_{\mathrm{on}}^{\mathrm{exhale}}$ for all treatment plans, a clinical treatment plan was adopted in cases where the clinical treatment plan used BA. If BA was not used in the clinical treatment plan, the gantry angle regions for BA were determined by referring to the liver in each patient. Table 1 shows the BA regions for all treatment plans except that of patient #3 (for whom structure‐based BA was adopted). Figure 4 illustrates the determined BA regions (blue dots), indicating each arc per patient from inside to outside. To generate $P_{\mathrm{off}}^{\mathrm{exhale}}$, we removed the gantry angle and structure BA functions from $P_{\mathrm{on}}^{\mathrm{exhale}}$. The optimization parameters, arc numbers, and normalization were same as those in the clinical treatment plans. Finally, one patient had two treatment plans, $P_{\mathrm{on}}^{\mathrm{exhale}}$ and $P_{\mathrm{off}}^{\mathrm{exhale}}$. Then, the primary CT images were the inspiratory CT images for the two treatment plans with and without BA, named $P_{\mathrm{on}}^{\mathrm{inhale}}$ and $P_{\mathrm{off}}^{\mathrm{inhale}}$, respectively. These plans were generated by recalculating dose distribution using the same treatment parameters of isocenter location, MU, rotational angles, and beam modulations. Reoptimization was not allowed. We confirmed that almost all the DVH metrics for $P_{\mathrm{on}}^{\mathrm{exhale}}$, $P_{\mathrm{off}}^{\mathrm{exhale}}$, $P_{\mathrm{on}}^{\mathrm{inhale}}$, and $P_{\mathrm{off}}^{\mathrm{inhale}}$ met the criteria shown in Table 2.

To evaluate the dosimetric impact of respiratory motion of the liver for PTV, the spinal cord, and the esophagus, these structures were precisely copied from clinical treatment plans that used expiratory CT images to those with inspiratory CT images without editing. Other organs such as the stomach, bowel, and liver were not considered in this study because the impact of their interfractional motion may be greater. The dose difference (*DD*) and relative value, *DD*%, were calculated using the following equations for both the $P_{\mathrm{on}}^{\mathrm{inhale}}$ versus $P_{\mathrm{on}}^{\mathrm{exhale}}$ plan and the $P_{\mathrm{off}}^{\mathrm{inhale}}$ versus $P_{\mathrm{off}}^{\mathrm{exhale}}$ plans. In both evaluations, the expiratory CT images were used as references.

(1) $$DD=D_{\mathrm{on}}^{\mathrm{inhale}}-D_{\mathrm{on}}^{\mathrm{exhale}}\;\mathrm{or}\;D_{\mathrm{off}}^{\mathrm{inhale}}-D_{\mathrm{off}}^{\mathrm{exhale}}$$

(2) $$\begin{array}{ccc} & & DD\%=100\times \left(\frac{D_{\mathrm{on}}^{\mathrm{inhale}}-D_{\mathrm{on}}^{\mathrm{exhale}}}{D_{\mathrm{on}}^{\mathrm{exhale}}}\right)\;\mathrm{or}\\ & & \;100\times \left(\frac{D_{\mathrm{off}}^{\mathrm{inhale}}-D_{\mathrm{off}}^{\mathrm{exhale}}}{D_{\mathrm{off}}^{\mathrm{exhale}}}\right) \end{array}$$

where, $D_{\mathrm{on}}^{\mathrm{inhale}}$ and $D_{\mathrm{off}}^{\mathrm{inhale}}$ are the DVH metrics from the $P_{\mathrm{on}}^{\mathrm{inhale}}$ and $P_{\mathrm{off}}^{\mathrm{inhale}}$ treatment plans, respectively; and $D_{\mathrm{on}}^{\mathrm{exhale}}$ and $D_{\mathrm{off}}^{\mathrm{exhale}}$ are the DVH metrics from the $P_{\mathrm{on}}^{\mathrm{exhale}}$ and $P_{\mathrm{off}}^{\mathrm{exhale}}$ treatment plans, respectively.

As illustrated in Figure 1b, we measured the longest and shortest points of the liver on the axial plane at the isocenter for both the inspiratory and expiratory CT images to examine the impact of respiratory motion of the liver. The size differences in each direction, $DL_{L}$ and $DL_{S}$, between the inspiratory and expiratory CT images were calculated as follows. If the liver disappeared at the axial plane in inspiratory CT images, $L_{L}^{\mathrm{inhale}}$ and $L_{S}^{\mathrm{inhale}}$ were zero. Similarly, the exhaled CT images were used as a reference.

(3) $$DL_{L}=L_{L}^{\mathrm{inhale}}-L_{L}^{\mathrm{exhale}}$$

(4) $$DL_{S}=L_{S}^{\mathrm{inhale}}-L_{S}^{\mathrm{exhale}}$$

(5) $$L_{\mathrm{ave}}=\frac{1}{2}\left(DL_{L}+DL_{S}\right)$$

Finally, averaged values for $L_{\mathrm{ave}}$ from the two difference sizes, $DL_{L}$ and $DL_{S}$, were used as indicators of the magnitude of liver movement. The correlation coefficients between *DD* and $L_{\mathrm{ave}}$ were determined. To ensure consistency in the liver size measurements, we used a function of MIM Maestro v.7.0.3 (Cleveland, OH, USA) that utilizes RECIST criteria whilst delineating liver volume. This study was approved by our institutional ethics review board (approval number: 2017−091) and conducted in accordance with the tenets of the 2013 revision of the Declaration of Helsinki.

### 2.3 Statistical analysis

The DVH metrics for the inspiratory and expiratory CT images were compared using the Wilcoxon signed‐rank test. The level of statistical significance was defined as a *p*‐value of < 0.05. Correlation strengths were determined according to correlation coefficient *r* ranges as follows: 0−0.19 = very weak, 0.2−0.39 = weak, 0.40−0.59 = moderate, 0.60−0.79 = strong, and 0.80−1.0 = very strong. All statistical analyses were performed using MATLAB 2022b (MathWorks, Natick, MA, USA).

## 3 RESULTS

**FIGURE 5:**
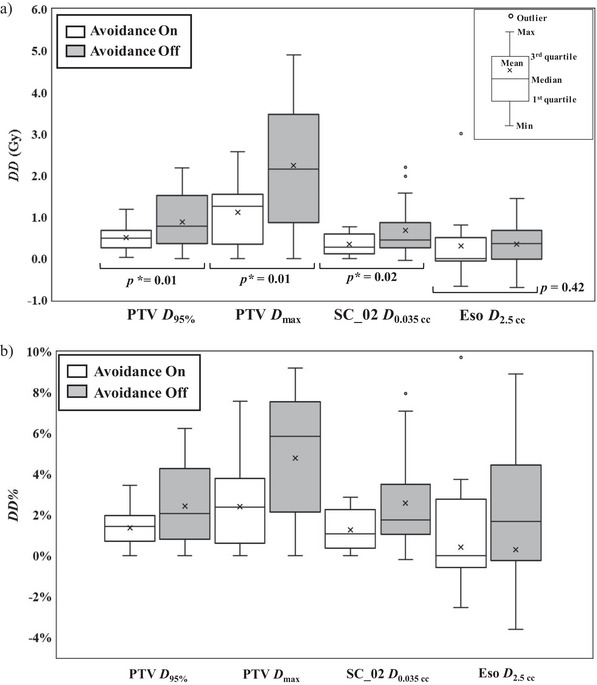
a) Dose difference *DD* and b) *DD*% in the DVH metrics of the SBRT treatment plans with and without beam avoidance. DVH, dose–volume histogram; SBRT, stereotactic body radiation therapy.

Figure 5 shows the differences in the DVH metrics, *DD* and *DD%* for the treatment plans with and without BA. The data in each case were provided in the Table S1. Expiratory CT as the primary CT images is utilized for dose calculation at our institution. Therefore, as shown in Equations (1) and (2), the DVH metrics from the exhaled CT images were used as the reference. During inhalation, the liver moved away from the target (Figure 1) producing negative $L_{\mathrm{ave}}$ values, while increased doses at the PTV, spinal cord, etc. results in a positive *DD* value. Significant differences were found between treatment plans with and without BA in PTV *D*_95%_, *D*_max_, and spinal cord *D*_0.035 cc_. Almost all DVH metrics for treatment plans with BA revealed smaller dose discrepancies in the delivered dose than those without BA.

**FIGURE 6:**
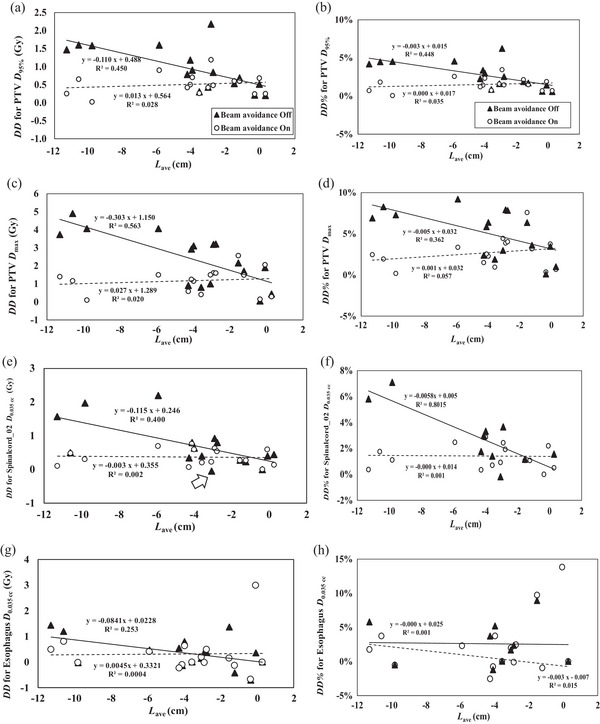
Relationships between a), c), e), g) *DD* and b), d), f), h) *DD*% in the DVH metrics and *L*_ave_ of the SBRT treatment plans with and without beam avoidance for the PTV, the spinal cord, and the esophagus. DVH, dose–volume histogram; SBRT, stereotactic body radiation therapy.

Figure 6 shows the relationships between *L*_ave_ and *DD* for the treatment plans with and without BA, displaying the regression curves applied to treatment plans with and without BA. Table 3 shows the correlation coefficients for the DVH metrics. Almost no significant correlations were found between DVH metrics for treatment plans with BA, indicating liver motion have little dosimetric impact on the plans with BA. However, those without BA showed moderate or strong correlations. For instance, for spinalcord_02, regarded as the most critical OAR, the *L*_ave_ of ≤−4 cm yields approximately 1 Gy or a +4% increase in dose.

**TABLE 3:** Correlation coefficients between *L*_ave_ and *DD* for spine metastasis radiation treatment plans with and without beam avoidance.

	*r*	*p*‐value	Correlation
With beam avoidance			
PTV *D*_95%_	0.00	0.99	Very weak
PTV *D*_max_	0.21	0.43	Weak
Spinalcord_02 *D*_0.035cc_	−0.12	0.66	Very weak
Esophagus *D*_2.5cc_	−0.17	0.54	Very weak
Without beam avoidance			
PTV *D*_95%_	−0.71	<0.01	Strong
PTV *D*_max_	−0.68	0.01	Strong
Spinalcord_02 *D*_0.035cc_	−0.56	0.02	Moderate
Esophagus *D*_2.5cc_	−0.45	0.10	Moderate

Abbreviations: *D*, dose; *L*_ave_, average liver size; DD, dose difference; PTV, planning target volume; spinalcord_02, spinal cord with a geometrical margin of 2 mm.

## 4 DISCUSSION

SBRT with VMAT for spine metastasis produces a conformal dose distribution with a steep dose gradient around the target. This can improve the dose coverage of the target and reduce unwanted doses to the OARs. In particular, adjacent organs such as the spinal cord have strict tolerance doses and excess dose carries a risk of radiation myelopathy.^9^, ^11^, ^14^ Significant discrepancies in the delivered dose can be induced due to patient setup inaccuracy during image guidance.^15^, ^16^ Other uncertainties include interobserver delineation errors, incorrect registrations, and inaccurate dose calculation. Such potential risk factors can be identified by multidisciplinary and systematic reviews of new advances in radiotherapy.^17^, ^18^ The present study clarified one of these uncertainties by focusing on the dosimetric impact of respiratory motion of the liver. Although this uncertainty occurs during irradiation and its effect may be smaller than those of factors such as patient setup and registration error, its investigation is necessary to optimize treatment accuracy in SBRT for spine metastasis.

This retrospective study revealed that 29 of 208 lesions (about 14%) patients with spine metastasis had metastasis near the liver dome, and 16 cases (subject of this study) shows Th7 and Th12 with most cases at Th10. Dose differences in the DVH metrics was demonstrated to be positive when using expiratory CT images as the primary CT in TPS because the liver as an attenuation medium does not appear during inhalation. This inferred that delivered doses can potentially be greater than planned doses if respiratory management measures such as breath‐holding are not performed. Treatment planning for spine metastasis SBRT is particularly difficult, and ensuring the planned spinal cord dose meets the $D_{0.035\;\mathrm{cc}}\leq$ 27.5 Gy constraint (as shown in Figure 3) can be problematic. Evaluation of patient level groups showed large discrepancies in the delivered dose, with increases from the planned dose of around 1 Gy (Figures 5a and 6e). This is equal to approximately 4% of the tolerance dose, which is not negligible. In such cases, BA should be used to mitigate discrepancies. Alternatively, patient can be asked to manage their respiratory functions through breath‐holding to reduce the effects of liver motion without applying BA. The effectiveness of respiratory management should be carefully weighed against its disadvantages, which include longer treatment time and increased patient burden.

The preferred practice at our institutions is to use expiratory CT images as the primary CT scan for TPS since before. On the other hand, these are usually free‐breathing CT images. However, the time required to scan the liver region or the time it takes for the treatment arc field to pass through the liver is relatively instantaneous. Moreover, the treatment fraction is small, so the delivered dose may vary and can depend on the patient. Accordingly, medical physicists need to know the dosimetric impact, and it is hoped that this investigation will provide some helpful insights into this.

As shown in Figure 5, significant differences in specific DVH metrics were observed between the treatment plans with and without BA. In almost all of the metrics, the *DD* was reduced by the introduction of BA. We have therefore demonstrated that BA is effective in mitigating the dosimetric impact of respiratory motion of the liver. In patients #3 and #4, the introduction of BA still yielded greater *DD* than other patients, whose spinal cord *DD* were 0.76 and 0.63 Gy, respectively. For patient #3, structure‐based BA was used. For patient #4, we adopted a narrow gantry angle range of 260–305° (red arrow in Figure 4) (the average range used in this study was 260–320°).

As shown in Figure 6, respiratory liver movement produced dosimetric changes in DVH metrics in the PTV and spinal cord, especially in treatment plans without BA (shown by solid triangle symbols). *DD* increased with increases in the absolute $L_{\mathrm{ave}}$ for PTV *D*_95_ and *D*_max_, and spinal cord *D*_0.035 cc_. Interestingly, less effect was observed for the esophagus. Even in cases where BA was not used and the absolute $L_{\mathrm{ave}}$ was large, *DD* was close to zero (indicated by the arrow in Figure 6e). This is because the inspiratory CT images showed that body thickness increased with inhalation, mitigating the effects of the dose increase. As observed in one case on the regression curve, if the absolute difference in liver size between the expiratory and inspiratory CT images is greater than 4 cm, the dosimetric impact of liver movement can be expected to be greater, resulting in a 1 Gy dose increase in the spinal cord dose. If inspiratory CT images are missing, *L*_ave_ can be calculated to evaluate the dosimetric impact before treatment, assuming that $L_{L}^{\mathrm{inhale}}$ and $L_{S}^{\mathrm{inhale}}$ are zero (i.e., the liver dome moves away from the target). This estimate would provide useful information for institution that only have inspiratory CT.

This study had several limitations. Indicators for different sizes were introduced on the isocentric plane between the expiratory and inspiratory CT images to evaluate the respiratory motion of the liver. These indicators may be useful as a simple value; however, it does not allow for three‐dimensional anatomical changes. In addition, only two phases of the expiratory and inspiratory CT images were examined, which did not represent actual respiratory motion of the patient. These results can likely be regarded as the maximum estimate. However, since the beam that passes through the liver with a rotational gantry is instantaneous, this estimation is not inappropriate as a guide. Additionally, we need to consider the optimum parameters rather than the *L*_ave_ proposed in this study. In principle, a water equivalent path between the skin and target for all arcs would be more meaningful. However, this is not an easy indicator to calculate, and its use would be impractical in clinical settings. Therefore, we proposed a simple *L*_ave_ metric to express respiratory changes in liver size.

## 5 CONCLUSION

Respiratory motion of the liver dome can potentially lead to dosimetric discrepancies of around 1 Gy in the delivered dose for the spinal cord *D*_0.035 cc_. Dose assessment should be performed to determine the appropriate form of respiratory management, such as breath‐holding. Alternatively, BA can effectively mitigate this effect.

## AUTHOR CONTRIBUTION

Midori Nonaka, Yuta Kobayashi, and Masato Nishitani gathered information the treatment planning system and analyzed the obtained data. Takahito Chiba, Satoshi Nakamura, Hiroki Nakayama, Kotaro Iijima, Tomoya Kaneda, Yoshihiro Shibata, Tomonori Goka, and Hiroshi Igaki discussed the study design before starting our research. Yasunori Shuto, Miki Yonemura, Riki Oshika, Hironori Kishida, Yuka Urago, Shuka Nishina, and Takumi Sakamoto provided some advice to prepare graphs and illustration in manuscript.

## CONFLICT OF INTEREST STATEMENT

There is no ethical problem or conflict of interest with regard to this manuscript. Okamoto H has a research grant by Elekta, Accuray, and Item Corporation. Igaki H has a research grant by Elekta.

## Supporting information

Supporting Information
