# Supplementary material for: Dosimetric impact of the respiratory motion of the liver dome in stereotactic body radiotherapy for spine metastasis: A planning study
Source: J Appl Clin Med Phys. 2024 Jul 1;25(9):e14403. doi: 10.1002/acm2.14403 (PMC11492324; doi:10.1002/acm2.14403)
Supplement: Supplementary file 1 — Supporting Information [file ACM2-25-e14403-s001.docx]

Supplementary Table 1. Dose differences *DD* for 16 spine metastasis cases.

| Case　# | *L*_ave_ (cm) | *DD* (Gy) | | | | | | | |
| --- | --- | --- | --- | --- | --- | --- | --- | --- | --- |
|  |  | Beam avoidance Off | | | | Beam avoidance On | | | |
|  |  | PTV *D*_95_ | PTV *D*_max_ | Spinalcord_02 *D*_0.035cc_ | Esophagus *D*_2.5cc_ | PTV *D*_95_ | PTV *D*_max_ | Spinalcord_02 *D*_0.035cc_ | Esophagus *D*_2.5cc_ |
| 1 | −3.6 | 0.3 | 0.8 | 0.4 | 0.0 | 0.3 | 0.4 | 0.2 | 0.0 |
| 2 | −4.0 | 0.9 | 3.1 | 0.6 | 0.8 | 0.7 | 1.2 | 0.6 | 0.6 |
| 3 | −4.1 | 1.2 | 2.9 | 0.8 | −0.1 | 0.5 | 1.2 | 0.8 | −0.1 |
| 4 | −2.9 | 2.2 | 3.2 | 0.9 | 0.4 | 1.2 | 1.6 | 0.6 | 0.0 |
| 5 | −3.1 | 0.4 | 1.0 | −0.1 | 0.2 | 0.4 | 1.5 | 0.2 | 0.2 |
| 6 | −1.5 | 0.5 | 2.2 | 0.3 | 1.4 | 0.6 | 2.6 | 0.3 | 0.1 |
| 7 | −4.3 | 0.8 | 0.9 | 0.3 | 0.5 | 0.4 | 0.6 | 0.1 | −0.2 |
| 8 | −11.3 | 1.5 | 3.7 | 1.6 | 1.4 | 0.3 | 1.4 | 0.1 | 0.5 |
| 9 | 0.3 | 0.2 | 0.4 | 0.4 | *N.A.* | 0.2 | 0.3 | 0.1 | *N.A.* |
| 10 | −9.8 | 1.6 | 4.1 | 2.0 | 0.0 | 0.0 | 0.1 | 0.3 | 0.0 |
| 11 | −10.6 | 1.6 | 4.9 | 0.5 | 1.2 | 0.7 | 1.2 | 0.5 | 0.8 |
| 12 | −0.1 | 0.5 | 1.9 | 0.4 | 0.4 | 0.7 | 2.1 | 0.6 | 3.0 |
| 13 | −1.2 | 0.7 | 1.7 | 0.2 | −0.4 | 0.6 | 1.5 | 0.3 | −0.1 |
| 14 | −0.4 | 0.2 | 0.1 | 0.0 | −0.7 | 0.2 | 0.2 | 0.0 | −0.7 |
| 15 | −2.8 | 0.8 | 3.2 | 0.8 | 0.4 | 0.5 | 1.6 | 0.5 | 0.5 |
| 16 | −5.9 | 1.6 | 4.1 | 2.2 | 0.5 | 0.9 | 1.5 | 0.7 | 0.4 |
| Mean$\pm$  SD | −4.1$\pm3.6$ | 0.9$\pm0.6$ | 2.4$\pm1.5$ | 0.7$\pm0.7$ | 0.4$\pm0.6$ | 0.5$\pm0.3$ | 1.2$\pm0.7$ | 0.4$\pm0.2$ | 0.3$\pm0.8$ |
| Median | −3.3 | 0.8 | 2.5 | 0.5 | 0.4 | 0.5 | 1.3 | 0.3 | 0.1 |
| Range | −11.3, 0.3 | 0.2, 2.2 | 0.1, 4.9 | −0.1, 2.2 | −0.7, 1.4 | 0.0, 1.2 | 0.1, 2.6 | 0.0, 0.8 | −0.7, 3.0 |

There were 15 patients but 16 cases as one patient had two lesions (cases 5 and 6). Ave, average; *D*, dose; *DD*, dose difference; Gy, Gray; *L*_ave_, average liver size; *NA*, organ did not exist near the dose delivery region; PTV, planning target volume; SD, standard deviation
